# Long-Term Surveillance of Food Products of Diverse Origins: A Five-Year Survey of Hepatitis A and Norovirus in Greece, 2019–2024

**DOI:** 10.3390/pathogens14020135

**Published:** 2025-02-02

**Authors:** Rafail Fokas, Zoi Anastopoulou, Kalypso-Angeliki Koukouvini, Maria-Eleni Dimitrakopoulou, Zoi Kotsiri, Eleftheria Chorti-Tripsa, Chrysoula Kotsalou, Dimosthenis Tzimotoudis, Apostolos Vantarakis

**Affiliations:** 1 Department of Public Health, Medical School, University of Patras, 26504 Patras, Greece; zoi.anastopoulou@gmail.com (Z.A.); k.koukouvini@gmail.com (K.-A.K.); dimitrakopoulou@upatras.gr (M.-E.D.); zoikotsiri@upatras.gr (Z.K.); chrysoulakotsalou@gmail.com (C.K.); 2 Department of Molecular Biology and Genetics, University of Thrace, 68100 Alexandroupolis, Greece; echorti@med.duth.gr (E.C.-T.); dtzimotoudis@gmail.com (D.T.)

**Keywords:** food safety, norovirus, Hepatitis A, surveillance, epidemiology, public health, Greece

## Abstract

This study examines at the prevalence and spread of Hepatitis A Virus (HAV) and norovirus GI/GII in local and imported food products in Greece over a five-year period (2019–2024). A total of two hundred sixty-six food samples were evaluated using obligatory inspections and virus detection procedures, including 202 for Hepatitis A and 64 for Norovirus. High-risk categories analyzed were vegetables [138 (HAV), 17 (NoV)], fruits [16 (HAV), 7 (NoV)], soft fruits/berries [37 (HAV), 31 (NoV)], processed meals [4 (HAV), 4 (NoV)], and animal-based products [1 (HAV), 5 (NoV)]. Viral RNA was isolated using QIAamp Viral RNA Mini Kit and detected using established RT-qPCR procedures that met ISO requirements for high sensitivity and reproducibility. The results demonstrated HAV contamination mostly in vegetables (4.35% positive rate), with sporadic findings in other categories. Norovirus GI/GII was detected primarily in soft fruits/berries, with a category-specific positive rate of 6.45%. A temporal study revealed that HAV peaks in 2020, while Norovirus contaminations were detected in 2021 and 2024. The findings highlight the important need to incorporate viral testing into routine food safety procedures, especially for high-risk product categories. This study establishes a basic framework for public health initiatives that address gaps in foodborne virus surveillance in Greece. The study’s ramifications extend to global efforts to monitor and reduce foodborne virus contamination, pushing for higher regulatory requirements and targeted preventative actions.

## 1. Introduction

Hepatitis A virus (HAV) and Norovirus (NoV) are significant public health viruses that share transmission modes and epidemic patterns but have unique etiologies. HAV, an acute liver infection spread through the fecal-oral route, is usually associated with food and waterborne infections, affecting millions worldwide due to different epidemiological patterns. Similarly, NoV, a primary cause of severe gastroenteritis, spreads quickly through contaminated food, water, and person-to-person contact, making it a major contributor to foodborne outbreaks [1].

HAV, a member of the Hepatovirus genus in the Picornaviridae family, is a major cause of acute hepatitis globally. HAV has five recognized genotypes, of which only I, II, and III infect humans [2]. The virus is primarily transmitted via the fecal-oral route and remains highly endemic in regions with poor sanitation, making it the leading cause of acute hepatitis worldwide [3,4]. Its remarkable resistance to high temperatures, acidity, and freezing enables it to persist in contaminated food, water, and surfaces for extended periods, contributing to its widespread transmission [5,6,7]. Transmission occurs directly through contact with infected individuals or indirectly via contaminated food and water, with recent outbreaks increasingly linked to imported frozen food and seafood [8,9,10,11].

HAV normally produces moderate, self-limiting sickness with lifelong immunity after infection [12] However, it can result in hospitalization and, in rare circumstances, death, especially in immunocompromised people [13,14,15]. Its low pathogen titter in food or human samples poses challenges for detection, making genomic epidemiology vital for outbreak tracking and surveillance [13,16]. 

Hepatitis A is tracked in Greece via the Mandatory Disease Declaration System, emphasizing the importance of attention. From 2004 to 2023, the National Public Health Organization (NPHO) recorded 1,985 cases, with an average of 99 cases per year (SD: 86) and a mean annual incidence of 0.91 cases per 100,000 people. Notably, 23.6% of cases were among Roma people, with youngsters under the age of 15 accounting for 88.9% of the total. Furthermore, 14.4% of instances were related to overseas travel, while 22.0% involved newly arrived or permanent migrants. Epidemics were observed among the Roma population in 2007 and 2013, recently arrived migrants in 2016, and men having sexual relations with men aged 25-44 in 2017 [17,18]. In the past decade, six positive samples were detected in foods. However, the majority of tracing efforts were based on clinical patient samples [18]. This underscores the importance of food tracing and reveals a significant gap in epidemiological surveillance. 

Norovirus is a tiny (27–40 nm) RNA virus from the Caliciviridae family with an RNA genome [19], and it is divided into ten genogroups (GI to GVX) and two unassigned groups based on gene sequences. Further classification identifies 49 capsid genotypes [20] and 60 p types [21], considerably expanding our understanding of norovirus variety. The GI, GII, GIV, and GIX are all harmful to humans [19,22]. GI and GII have 9 and 29 genotypes, respectively, with GII.4 being the most common strain worldwide [19,23]. Owing to its genetic and antigenic diversity, developing long-lasting immunity against norovirus or producing effective vaccines is challenging [24]. NoV is a leading cause of acute gastroenteritis cases and outbreaks worldwide among people of all ages [25]. While the most reported route is via direct person-to-person transmission, human noroviruses can be readily transmitted by food and water [26].

Various bacterial, viral, and protozoan pathogens induce gastrointestinal infections, and norovirus genotypes I (GI) and II (GII) are the major viral pathogens [27]. During the COVID-19 pandemic, norovirus has remained the leading cause of gastrointestinal diseases in South Korea, despite a reduction in various viral and bacterial infections [28]. An estimated 125 million cases of foodborne illness and 35,000 deaths globally have been attributed to norovirus [29]. The US Centers for Disease Control and Prevention (CDC) estimate that it is the most common cause of acute gastroenteritis in the United States with 21 million cases each year and an estimated 70,000 hospitalizations and 8000 deaths nationwide [30]. It is estimated that over 1.8 million children (under 5 years old) die from complications of NoV infection worldwide annually [31].

Recent unpublished epidemiological data indicate Norovirus as a continuous public health hazard in Greece. According to the National Public Health Organization, unconfirmed Norovirus cases were reported in Greece and adjacent Greek cities between October and November 2024. This outbreak highlights the virus’s high transmissibility and potential to create severe public health issues, underlining the importance of targeted surveillance and control efforts to reduce the hazards associated with foodborne viral infections.

This study aims to address the critical gap in understanding the prevalence and distribution of Hepatitis A Virus (HAV) and norovirus GI/GII in both domestically produced and imported food products in Greece. By analyzing food samples collected over a five-year period, this research seeks to identify high-risk food categories and explore temporal trends and geographic distribution of contamination. This study highlights the necessity of integrating viral testing into routine food safety monitoring systems, particularly for high-risk products. Furthermore, the findings will provide valuable insights to support the development of targeted interventions and improve public health outcomes, underlining the significance of comprehensive foodborne virus surveillance in Greece.

## 2. Materials and Methods

### 2.1. Sample Collection

A total number of two hundred sixty-six food samples (202 [HAV], 64 [NoV]) were collected as part of mandatory inspections by governmental entities, including customs offices, to guarantee compliance with health and safety regulations for 5 consecutive years (2019–2024). Samples from other countries, especially outside the European Union, were tested up to 5% of the imported ones according to EU guidulines, to assess the potential risk of norovirus and Hepatitis A contamination. Samples from Greek local enterprises were also included in the study to guarantee complete surveillance of viral contamination in food products throughout the country.

**Figure 1:**
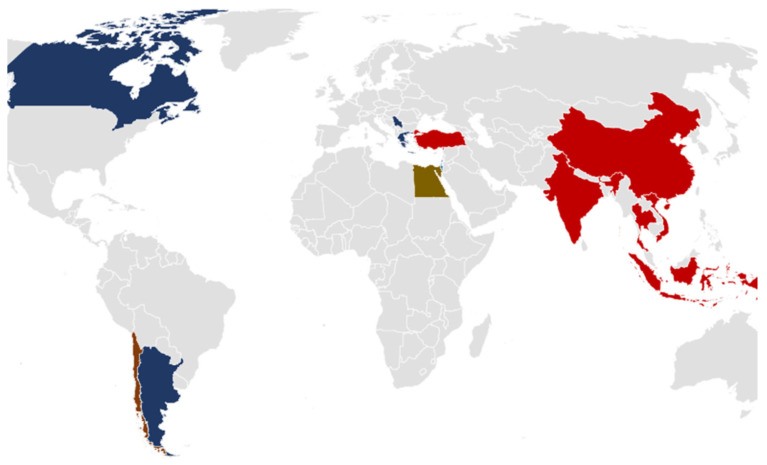
Geographic distribution of food origins.

The samples were chosen using public health criteria, with a priority on imported products from high-risk regions while considering domestic food safety requirements. Figure 1 depicts the geographic distribution of origin countries, encompassing regions within Greece and countries of origin for imported food goods. Most of the samples originated from Turkey and Greece. The samples were collected and received within 24 h, either in freezing conditions for frozen products or in refrigerated conditions (±5 °C) for fresh products, except for dried or preserved in oil products, and were forwarded for analysis.

### 2.2. Categorization of Food Samples

The food samples analyzed in this study were carefully classified based on their type (Table 1) and preparation method to evaluate viral contamination trends and possible dangers across various food groups. This categorization clarifies and helps identify high-risk food categories for HAV and NoV GI/GII. Detailed information regarding the number of samples analyzed, their geographic origins, and the years of collection for each food category and virus type is provided in the supplementary material (Table S1).

**Figure 2:**
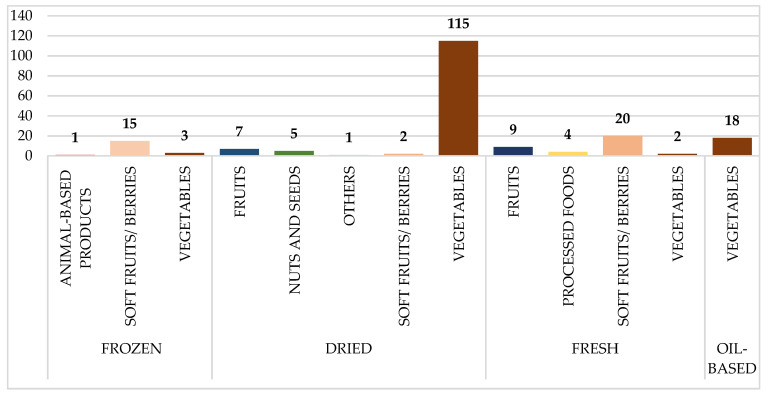
Distribution of analyzed food samples tested for Hepatitis A (HAV), categorized by food type and preparation method. Numbers on the bars represent the total number of samples collected per category.

Figure 2 depicts the distribution of HAV-tested food samples, broken down by food type and preparation method. Dried veggies are the largest group, with 115 samples. Other substantial contributions include soft fruits/berries (20 fresh and 15 frozen samples) and oil-based veggies (18 samples). Animal-based and processed foods were underrepresented, with only a few samples provided.

**Figure 3:**
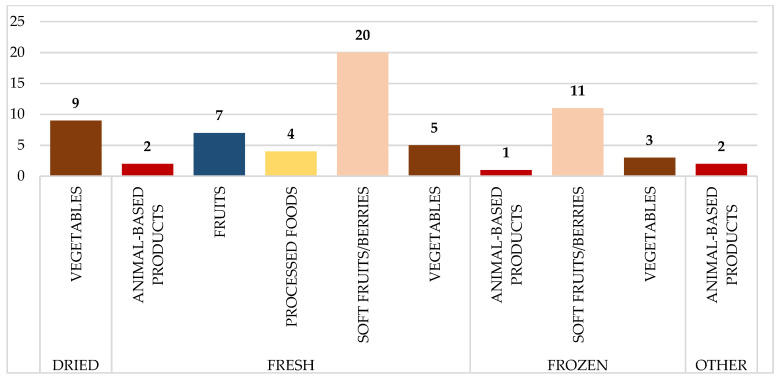
Distribution of analyzed food samples tested for norovirus GI/GII, categorized by food type and preparation method. Numbers on the bars represent the total number of samples collected per category.

Figure 3 depicts the distribution of food samples tested for Norovirus GI/GII, which is further categorized by food type and preparation method. The fresh soft fruits/berries category received the most samples (20), followed by frozen soft fruits/berries (11) and dry vegetables (9). Animal-based products and processed foods, on the other hand, contributed insignificantly, with few samples across preparation styles.

Food samples were chosen based on importation patterns and national risk assessment laws, which reflect Greece’s epidemiological surveillance priorities. While the distribution varies by food category and origin, it is consistent with the regulatory framework and public health objectives.

### 2.3. Virus Concentration and Extraction

The virus concentration and extraction procedure were based on the ISO/TS 15216-1:2013. The process of concentrating viruses from food matrices is a critical step in the detection of viral contaminants such as hepatitis A virus and norovirus. Given the complexity and variability of food matrices, specific methods are tailored to different food types to optimize virus recovery. For soft fruits and salad vegetables, viruses are extracted through an elution process using Tris/glycine/beef extract buffer (TGBE) with agitation, followed by precipitation with polyethylene glycol (PEG) and sodium chloride. All buffers and reagents were prepared in-house according to standardized laboratory protocols. Animal products require enzymatic digestion using proteinase K (Macherey-Nagel, Düren, Germany) to release viral particles, followed by centrifugation to isolate the concentrated sample. Extraction of viral RNA was performed using QIAamp Viral RNA Mini Kit (Qiagen, Hilden, Germany) following the procedure based on the instructions provided by the manufacturer.

### 2.4. One Step RT-PCR for HAV, NoV GI and GII

An RT-qPCR assay was performed using Thermocycler Stratagene Mx30005P (Thermo Scientific, Waltham, MA, USA). The assay was performed according to the recommendations of the manufacturer. The protocol used is a TaqMan-based real-time RT-PCR (qPCR) for the qualitative and quantitative detection of noroviruses GI and GII and for Hepatitis A using RNA UltraSense™ One-Step Quantitative RT-PCR System (Applied Biosystems™, Thermo Fisher Scientific, Waltham, MA, USA), based on ISO/TS 15216-1:2013 with slight modifications. Standard curves used in qPCR were generated by using serial dilutions of known amounts of genome copies of the targeted genes. To guarantee the quality of the assay, each run included a non-template control-negative control (RNase-free grade water), a positive control, a positive process control using Mengo virus Extraction Control kit (Biomeriex, Craponne, France), and a negative process control, all in duplicates. Each sample was run in duplicates and in tenfold dilutions for the assessment of the PCR reaction inhibition. The LOD of the assay was determined at 10 genome copies per reaction. Primer sets are described in Table 2. The reaction took place in a final reaction volume of 25 μL, with 10 μL of sample in a 96-well optical reaction plate. Cycling conditions were 15 min at 50 °C for reverse transcription, 2 min at 95 °C for enzyme activation, 50 cycles of 15 s at 95 °C, and 1min at 60 °C for annealing and extension (45 cycles). Efficiency for all targets was determined based on the equation Efficiency (e%) = E = 10^(−1/slope)^ − 1 [32]. For Hepatitis A assay, e% = 96.5, Slope = −3.408, y-intercept = 36.49, and Rsq = 0.995. For norovirus GI assay, e% = 96.5, Slope = −3.410, y-intercept = 42.31, and Rsq = 0.999. For norovirus GII assay, e% = 97.3, Slope = −3.389, y-intercept = 39.24, and Rsq = 0.998. Results were expressed as the Presence of “x” virus, “x” detectable genome copies per gr of food mat rice, or no Presence.

### 2.5. Validation of Analytical Procedures

The validation of analytical procedures ensures the reliability and accuracy of detecting viral RNA in food samples through a structured and rigorous approach. It includes constructing standard curves with a minimum of three dilutions, requiring a Pearson correlation coefficient (r^2^) of at least 0.98 to confirm the linearity of amplification. Amplification efficiency is assessed by comparing quantification cycle (Cq) values between controls and test samples, with strict thresholds for determining valid results. Extraction efficiency is calculated, ensuring the recovery rate meets the protocol’s criteria. Additionally, the protocol defines both the theoretical and practical limits of detection, specifying minimum detectable quantities of viral RNA based on sample type and preparation methods. Controls, including negative and external RNA standards, are integral to identifying inhibition or contamination during testing. This thorough validation framework maintains high standards of reproducibility and accuracy, essential for the reliable qualitative detection of target viral genomes.

### 2.6. Statistical Analysis

The data collected for this study were organized and analyzed using Microsoft Excel. Samples were divided into predetermined categories based on food type (e.g., fruits, vegetables, processed foods) (Figure 2 and Figure 3) and viral infection status. Subgroups were further divided by country of origin and year of sampling, allowing for a thorough examination of temporal trends and geographic dispersion. To determine virus prevalence, the statistical analysis involved calculating the percentage of positive samples as well as 95% confidence intervals (CIs). Descriptive statistics were used to assess the distribution of positive samples among categories.

## 3. Results

Of the total number of positive samples for Hepatitis A, four (50%) were found to be positive from Greece and four (50%) were found to be positive from Turkey. As for Norovirus, all four (100%) positive samples came from Greece (Table 3). 

The analysis found a significant predominance of vegetable samples, accounting for most of the dataset for HAV detection. Vegetables had a positive rating of 4.35% within the category, adding up to a total of 2.97% positive across all samples (Table 4). Soft fruits and berries were also identified as a substantial group, accounting for 18.32% of all samples examined, although having a lower positive rate of 2.7% within the category. Animal-based goods, which accounted for only 0.5% of the total samples, stood out due to their extraordinarily high category-specific positivity rate of 100%, indicating a single positive sample. Other categories, such as fruits, processed foods, nuts and seeds, and others, produced no positive samples, indicating that these groupings were not contaminated with Hepatitis A.

Soft fruits/berries constituted the majority of samples (48.44%) and showed a 6.45% positivity rate within the category, corresponding to an overall positivity rate of 3.12% (Table 5). Vegetables, which made up 26.56% of the samples, exhibited no signs of Norovirus GI/GII infection. Fruits and processed foods performed particularly well in minor categories. Fruits accounted for 10.94% of the samples and had a category-specific positivity rate of 14.29%, but processed foods, which accounted for just 6.25% of the total samples, had the highest positivity rate within the category at 25%. Nuts and seeds, animal-based goods, and other categories produced no positive samples, indicating that contamination did not exist.

The Table 6 summarizes the prevalence of viral contamination in the tested samples, focusing on the overall positivity rates for Hepatitis A and norovirus GI/GII. Hepatitis A was discovered in 8 of 202 samples, accounting for 3.96% positive. The viral type of prevalence in the samples studied ranged between 1.3% and 6.6%. For norovirus GI/GII, four out of 64 samples tested positive, resulting in a 6.25% positivity rate. The virus type prevalence for norovirus GI/GII was estimated at 0.3–12.1%, indicating a broader confidence interval due to the small sample number.

The positive cases for HAV and NoV were documented to provide an overview of their distribution during the study period. Positive HAV samples were detected in December 2019, March 2020, May 2020, August 2020, October 2020, and November 2020. Similarly, Norovirus-positive samples were identified in April 2021, July 2021 and March 2024.

## 4. Discussion

Semi-dried tomatoes have been connected to Hepatitis A Virus outbreaks in Australia, the Netherlands, and England, highlighting the significance of foodborne transmission in the virus’s spread [37,38]. This recurring association has made semi-dried tomatoes a critical focus in our study, representing a significant portion of the analyzed vegetable samples. The regulatory emphasis on monitoring high-risk categories further highlights the need for targeted surveillance of such items, particularly those sourced from endemic regions like Turkey [39] and Egypt [40]. 

Globally, HAV outbreaks have been reported in various regions, including in Thailand [41], India (2004), Philippines and Vietnam (2011–2017) [42], Egypt (2013–2014) [40], Malaysia (2012) [43], Taiwan (2015–2017) [44], Indonesia (2015–2016) [45], and the United States (2017–2018) [46]. While vaccination programs and improved sanitation have significantly reduced HAV cases in developed countries [47] outbreaks continue to be reported in both developed and developing regions. According to the latest data published from the European Centre for Disease Control and Prevention, the average reported cases in European Union countries (excluding the UK) in 2023 was 6199 [48].

Surveillance data from Greece highlight a persistent low reported incidence of hepatitis A, with a rate of 0.08 cases per 100,000 population in 2023—significantly lower than the average 1.00 cases per 100,000 observed in European Union (EU) countries [48]. The reported drop in hepatitis A cases in Greece since 2019 could be due to a mix of structural improvements and external causes. Enhanced sanitation, improved water quality, and efficient immunization programs for children and high-risk groups have all lowered transmission risks. Furthermore, the COVID-19 pandemic most likely influenced these patterns through increased hygiene standards and disruptions to healthcare access, which may have slowed case detection or contributed to underreporting [49]. Despite these gains, the possibility of new outbreaks remains, particularly in populations with poor vaccination rates or inadequate sanitary infrastructure.

Importantly, findings from this study revealed no HAV-positive samples from 2021 onward, faithfully following the reduced case rates as reported by the ECDC [48]. Among the eight HAV-positive samples identified, four originated from domestically produced food, while the remaining four were traced to imported products, specifically from Turkey. Turkey continues to have an intermediate to high prevalence of Hepatitis A among its population [39,50]. Notably, vegetables showed a positivity rate of 4.35%, while soft fruits and berries exhibited a rate of 2.70%, underscoring the importance of monitoring these high-risk food categories. These findings apply to two types of food that are often collected by hand by workers, making contamination via a vector possible. Migrant workers play a significant role in Greek agriculture, particularly in the harvesting and production of fruits and vegetables, which are key components of the food supply chain [51]. Many migrants have been tested positive for hepatitis A in communal buildings and housing facilities in Greece [52]. This demographic’s engagement in food-related activities emphasizes the importance of effective surveillance and public health measures to reduce possible contamination hazards. The equal distribution of HAV-positive samples between domestic and imported goods underscores the need for comprehensive quality control measures. These should focus both on reducing risks during harvest—especially in produce collected by hand, which can be susceptible to contamination from infected workers—and on ensuring that contamination does not occur at later stages of food processing or transportation, regardless of the origin of the products. Addressing suboptimal hygiene practices and ensuring equitable access to healthcare and vaccination [53] will be critical to mitigating transmission risks.

On the other hand, Norovirus outbreaks linked to the consumption of bivalve mollusks impacted by contaminated waters are well-documented [54,55,56]. Studies have reported prevalence rates in shellfish ranging from nil to approximately 70%, with higher detection rates observed in the UK (68.7%) [57] and the EU (34.5%) [58]. Lower rates, such as <2% in Australia [59], 3.9% in the US [60], 12.3% (37/300) in Thailand [61] highlight regional differences in production environments and management practices.

Τhe prevalence of norovirus in Greece is not well monitored, owing to the virus’s nature and the lack of strict reporting requirements, as hospitalization is not always required. Consequently, the exact number of cases remains unknown. However, outbreaks have been observed, including one in northeastern Greece in June 2006 [62] and another on a remote island in the Northern Aegean Sea in February 2010, both of which were connected to seafood eating [63]. More recently, in the fall of 2024, an unregistered increase in norovirus cases was observed in Crete, Greece, likely attributed to heightened tourist activity.

The study’s findings revealed that all four norovirus-positive samples originated from domestically produced food products in Greece. Notably, soft fruits and berries accounted for two of these positive cases, with the remaining two identified in fruits and processed foods. The positive rate for soft fruits and berries was 6.45%, whereas vegetables had a rate of 0%. Fruits made up 10.94% of the total samples, with a category-specific positive rate of 14.29%. Processed foods, accounting for only 6.25% of total samples, had the highest category-specific positive rate of 25%. As with Hepatitis A, the categories of positive norovirus tests relate to foods collected by hand, which increases the chance of infection.

One notable weakness of this study is the minimal number of positive samples de-tected, which limits the statistical validity of the conclusions. Sampling bias, particularly in food categories with a little market presence, might have impacted on the findings. Furthermore, the lack of thorough data on norovirus occurrence in Greece and elsewhere makes it difficult to adequately assess its public health impact. Despite these limitations, this study emphasizes the significance of ongoing monitoring and increased collaboration among stakeholders in guaranteeing food safety. The study emphasizes the importance of investing in improved detection technology and establishing strong control measures to reduce the danger of viral transmission via food. Setting strict quality standards and implementing targeted public health interventions is especially important for high-risk food categories and vulnerable groups, such as Roma communities and migrant workers who play important roles in food production and processing. Integrating epidemiological surveillance of clinical cases into national health systems is also critical for improving traceability and gaining a thorough understanding of viral transmission patterns.

Foodborne spread of hepatitis A and norovirus underscores the importance of strict food safety standards. To reduce risk, comprehensive screening systems should be implemented for both domestic and imported high-risk food products. Sustained investment in monitoring systems, public education efforts, and strong food safety legislation will protect public health and the integrity of Greece’s food sector. This work addresses these difficulties, providing vital information to improve food supply chain safety and inform public health choices.

## 5. Conclusions

This study emphasizes the need of incorporating foodborne virus surveillance into national food safety systems. The findings show the prevalence of Hepatitis A and Norovirus in high-risk food categories, including vegetables, soft fruits, and processed foods, emphasizing the importance of tight quality control procedures. The identification of HAV and Norovirus-positive samples in both local and imported food products emphasizes the significance of maintaining a balanced surveillance approach that takes into account epidemiological risk factors and trade trends. While the limited number of positive samples gathered may have an impact on the study’s statistical soundness, the findings give critical insights into the vulnerabilities in the food supply chain. Sampling bias due to market availability and product categories was acknowledged, but the study design is consistent with Greece’s regulatory framework and epidemiological aims. To improve foodborne virus surveillance, we advocate strengthening sample procedures, broadening the scope to incorporate clinical case data, and deploying integrated monitoring systems.

## Figures and Tables

**Table 1:** Categorization of analysed food samples based on Food Type.

Food Category	Type of Food
Vegetables	Okra, Sun-Dried Tomatoes, Garlic, Tomatoes, Salad, Lettuce, Spinach
Fruits	Plums, Dates, Cherries, Apricots, Processed Fruits, Fruits, Mango
Soft Fruits/Berries	Raspberries, Strawberries, Fragostafylla, Berries, Blueberries, Cranberries, Soft Fruits
Animal-Based Products	Mussels
Nuts and Seeds	Peanuts, Hazel, Cassius
Processed Foods	Sun-Dried Tomatoes (Paste), Tomato (Paste), Sauces
Others	Cinnamon

**Table 2:** Oligonucleotide primer and probe sets Hepatitis A, norovirus GI and GII.

Viruses	Primers and Probes	Sequence	References
Hepatitis A	HAV68 (Fw)	TCACCGCCGTTTGCCTAG	[33]
	HAV (Rv)	GGAGAGCCCTGGAAGAAAG	
	HAV150 (Probe)	[FAM]-CCTGAACCTGCAGGAATTAA-[BHQ1]	
Norovirus GI	QNIF4 (Fw)	CGCTGGATGCGNTTCCAT	[34]
	NV1LCR (Rv)	CCTTAGACGCCATCATCATTTAC	
	NVGG1p (Probe)	[FAM]-TGGACAGGAGAYCGCRATCT-[TAMRA]	
Norovirus GII	QNIFS (Fw)	ATGTTCAGRTGGATGAGRTTCTCWGA	[35,36]
	COG2R (Rv)	TCGACGCCATCTTCATTCACA	
	QNIFs (Probe)	[FAM]-AGCACGTGGGAGGGCGATCG-[TAMRA]	

**Table 3:** Summary of positive food samples (HAV and NoV) detected during the study period.

Type Of Food	Category	Kind	Origin	Month	Year	Virus
Okra	Vegetables	Frozen	Greece	December	2019	HAV
Okra	Vegetables	Frozen	Greece	December	2019	HAV
Mussels	Animal-Based Products	Frozen	Greece	March	2020	HAV
Sun-Dried Tomatoes	Vegetables	Dried	Turkey	May	2020	HAV
Sun-Dried Tomatoes	Vegetables	Dried	Turkey	August	2020	HAV
Sun-Dried Tomatoes	Vegetables	Dried	Turkey	October	2020	HAV
Sun-Dried Tomatoes	Vegetables	Dried	Turkey	October	2020	HAV
Soft Fruits	Soft Fruits/ Berries	Frozen	Greece	November	2020	HAV
Strawberries	Soft Fruits/Berries	Fresh	Greece	April	2021	NoV
Strawberries	Soft Fruits/Berries	Fresh	Greece	July	2021	NoV
Cherry	Fruits	Fresh	Greece	July	2021	NoV
Sun-Dried Tomatoes (Paste)	Processed Foods	Fresh	Greece	March	2024	NoV

**Table 4:** Data showing the number of samples collected according to single food type and their respective percentages for presence of Hepatitis A.

Category	Total Samples	% Whole Sampling	Positive Samples	% Positivity per Category	% Positivity Whole Sampling
Vegetables	138	68.32	6	4.35	2.97
Fruits	16	7.92	0	0	0
Soft fruits/Berries	37	18.32	1	2.70	0.5
Processed foods	4	1.98	0	0	0
Nuts and Seeds	5	2.48	0	0	0
Animal Based products	1	0.5	1	100	0.5
Others	1	0.5	0	0	0

The total number of samples analyzed for Hepatitis A was 202 samples.

**Table 5:** Data showing the number of samples collected according for each food type and their respective collection percentages for Norovirus GI/GII.

Category	Total Samples	% Whole Sampling	Positive Samples	% Positivity per Category	% Positivity Whole Sampling
Vegetables	17	26.56	0	0	0
Fruits	7	10.94	1	14.29	1.56
Soft fruits/Berries	31	48.44	2	6.45	3.12
Processed foods	4	6.25	1	25	1.56
Nuts and Seeds	0	0	0	0	0
Animal Based products	5	7.81	0	0	0
Others	0	0	0	0	0

The total number of samples analyzed for norovirus GI/GII was 64 samples.

**Table 6:** Number of positive tests and prevalence of HAV and norovirus GI/GII in collected samples.

Virus	Positive Tests	% Positive Tests	Virus Type Prevalence	Total Samples
Hepatitis A	8	3.96	1.3–6.6	202
Norovirus GI/GII	4	6.25	0.3–12.1	64

## Data Availability

Data are contained within the article.

## Supplementary Materials

The following supporting information can be downloaded at: https://www.mdpi.com/article/10.3390/pathogens14020135/s1, Table S1: Detailed Summary of Analyzed Food Samples: Number of Samples, Geographic Origin, Year of Collection, and Virus Detection (HAV and NoV).
