# Supplementary material for: Long-Term Surveillance of Food Products of Diverse Origins: A Five-Year Survey of Hepatitis A and Norovirus in Greece, 2019–2024
Source: Pathogens. 2025 Feb 2;14(2):135. doi: 10.3390/pathogens14020135 (PMC11857987; doi:10.3390/pathogens14020135)
Supplement: Supplementary file 1 [file pathogens-14-00135-s001.zip › pathogens-3428626-supplementary Table S1.pdf]

Sample No	Type Of Food	Type Of Food Categories	Kind	Origin	Origin Categories	Order Date	Year	HAV
**1**	Okra	Vegetables	Frozen	Greece	Europe	18/12/2019	2019	Presence
**2**	Okra	Vegetables	Frozen	Greece	Europe	18/12/2019	2019	Presence
**3**	Plums	Fruits	Dried	Chile	Other	30/1/2020	2020	Absence
**4**	Raspberries	Soft Fruits/ Berries	Frozen	Greece	Europe	6/2/2020	2020	Absence
**5**	Raspberries	Soft Fruits/ Berries	Frozen	Greece	Europe	6/2/2020	2020	Absence
**6**	Raspberries	Soft Fruits/ Berries	Frozen	Greece	Europe	10/2/2020	2020	Absence
**7**	Raspberries	Soft Fruits/ Berries	Frozen	Greece	Europe	11/2/2020	2020	Absence
**8**	Raspberries	Soft Fruits/ Berries	Frozen	Greece	Europe	10/2/2020	2020	Absence
**9**	Mussels	Animal-Based Products	Frozen	Greece	Europe	10/3/2020	2020	Presence
**10**	Strawberries	Soft Fruits/ Berries	Fresh	Greece	Europe	30/3/2020	2020	Absence
**11**	Okra	Vegetables	Frozen	Greece	Europe	27/4/2020	2020	Absence
**12**	Sun-Dried Tomatoes	Vegetables	Dried	Turkey	Asia	18/5/2020	2020	Absence
**13**	Sun-Dried Tomatoes	Vegetables	Dried	Turkey	Asia	18/5/2020	2020	Presence
**14**	Raspberries	Soft Fruits/ Berries	Frozen	Greece	Europe	25/5/2020	2020	Absence
**15**	Strawberries	Soft Fruits/ Berries	Frozen	Egypt	Africa	7/7/2020	2020	Absence
**16**	Sun-Dried Tomatoes	Vegetables	Dried	Turkey	Asia	17/8/2020	2020	Presence
**17**	Sun-Dried Tomatoes	Vegetables	Dried	Turkey	Asia	28/8/2020	2020	Absence
**18**	Sun-Dried Tomatoes	Vegetables	Dried	Turkey	Asia	8/9/2020	2020	Absence
**19**	Sun-Dried Tomatoes	Vegetables	Dried	Turkey	Asia	16/9/2020	2020	Absence
**20**	Sun-Dried Tomatoes	Vegetables	Dried	Turkey	Asia	17/9/2020	2020	Absence
**21**	Sun-Dried Tomatoes	Vegetables	Dried	Turkey	Asia	16/9/2020	2020	Absence
**22**	Raspberries	Soft Fruits/ Berries	Frozen	Greece	Europe	21/9/2020	2020	Absence
**23**	Fragostrafylla	Soft Fruits/ Berries	Frozen	Greece	Europe	22/9/2020	2020	Absence
**24**	Fragostrafylla	Soft Fruits/ Berries	Frozen	Greece	Europe	22/9/2020	2020	Absence
**25**	Sun-Dried Tomatoes	Vegetables	Dried	Turkey	Asia	24/9/2020	2020	Absence
**26**	Sun-Dried Tomatoes	Vegetables	Dried	Turkey	Asia	29/9/2020	2020	Absence
**27**	Sun-Dried Tomatoes	Vegetables	Dried	Turkey	Asia	29/9/2020	2020	Absence
**28**	Sun-Dried Tomatoes	Vegetables	Dried	Turkey	Asia	29/9/2020	2020	Absence
**29**	Sun-Dried Tomatoes	Vegetables	Dried	Turkey	Asia	29/9/2020	2020	Absence
**30**	Dates	Fruits	Dried	Turkey	Asia	28/9/2020	2020	Absence
**31**	Sun-Dried Tomatoes	Vegetables	Dried	Turkey	Asia	19/10/2020	2020	Absence
**32**	Sun-Dried Tomatoes	Vegetables	Dried	Turkey	Asia	19/10/2020	2020	Absence
**33**	Sun-Dried Tomatoes	Vegetables	Dried	Turkey	Asia	26/10/2020	2020	Absence
**34**	Sun-Dried Tomatoes	Vegetables	Dried	Turkey	Asia	30/10/2020	2020	Presence
**35**	Sun-Dried Tomatoes	Vegetables	Dried	Turkey	Asia	30/10/2020	2020	Presence
**36**	Soft Fruits	Soft Fruits/ Berries	Frozen	Greece	Europe	3/11/2020	2020	Presence
**37**	Sun-Dried Tomatoes	Vegetables	Dried	Turkey	Asia	4/11/2020	2020	Absence
**38**	Sun-Dried Tomatoes	Vegetables	Dried	Turkey	Asia	9/11/2020	2020	Absence
**39**	Sun-Dried Tomatoes	Vegetables	Dried	Turkey	Asia	10/11/2020	2020	Absence
**40**	Sun-Dried Tomatoes	Vegetables	Dried	Turkey	Asia	12/11/2020	2020	Absence
**41**	Sun-Dried Tomatoes	Vegetables	Dried	Turkey	Asia	11/11/2020	2020	Absence
**42**	Sun-Dried Tomatoes	Vegetables	Dried	Turkey	Asia	18/11/2020	2020	Absence
**43**	Sun-Dried Tomatoes	Vegetables	Dried	Turkey	Asia	23/11/2020	2020	Absence
**44**	Sun-Dried Tomatoes	Vegetables	Dried	Turkey	Asia	1/12/2020	2020	Absence
**45**	Sun-Dried Tomatoes	Vegetables	Dried	Turkey	Asia	1/12/2021	2020	Absence
**46**	Sun-Dried Tomatoes	Vegetables	Dried	Turkey	Asia	1/12/2020	2020	Absence
**47**	Sun-Dried Tomatoes	Vegetables	Dried	Turkey	Asia	14/12/2020	2020	Absence
**48**	Sun-Dried Tomatoes	Vegetables	Dried	Turkey	Asia	23/12/2020	2020	Absence
**49**	Sun-Dried Tomatoes	Vegetables	Dried	Turkey	Asia	7/1/2021	2021	Absence
**50**	Sun-Dried Tomatoes	Vegetables	Dried	Turkey	Asia	18/1/2021	2021	Absence
**51**	Sun-Dried Tomatoes	Vegetables	Dried	Turkey	Asia	27/1/2021	2021	Absence
**52**	Plums	Fruits	Dried	Chile	America	2/2/2021	2021	Absence
**53**	Sun-Dried Tomatoes	Vegetables	Dried	Turkey	Asia	8/2/2021	2021	Absence
**54**	Sun-Dried Tomatoes	Vegetables	Dried	Turkey	Asia	3/2/2021	2021	Absence
**55**	Sun-Dried Tomatoes	Vegetables	Dried	Turkey	Asia	17/3/2021	2021	Absence

**56**	Sun-Dried Tomatoes	Vegetables	Dried	Turkey	Asia	17/3/2021	2021	Absence
**57**	Sun-Dried Tomatoes	Vegetables	Dried	Turkey	Asia	17/3/2021	2021	Absence
**58**	Sun-Dried Tomatoes	Vegetables	Dried	Turkey	Asia	23/3/2021	2021	Absence
**59**	Sun-Dried Tomatoes	Vegetables	Dried	Turkey	Asia	26/3/2021	2021	Absence
**60**	Sun-Dried Tomatoes	Vegetables	Dried	Turkey	Asia	5/4/2021	2021	Absence
**61**	Sun-Dried Tomatoes	Vegetables	Dried	Turkey	Asia	7/4/2021	2021	Absence
**62**	Strawberries	Soft Fruits/ Berries	Fresh	Greece	Europe	12/4/2021	2021	Absence
**63**	Sun-Dried Tomatoes	Vegetables	Dried	Turkey	Asia	6/5/2021	2021	Absence
**64**	Sun-Dried Tomatoes	Vegetables	Dried	Turkey	Asia	6/5/2021	2021	Absence
**65**	Sun-Dried Tomatoes	Vegetables	Dried	Turkey	Asia	6/5/2021	2021	Absence
**66**	Sun-Dried Tomatoes	Vegetables	Dried	Turkey	Asia	28/5/2021	2021	Absence
**67**	Sun-Dried Tomatoes	Vegetables	Dried	Turkey	Asia	4/6/2021	2021	Absence
**68**	Sun-Dried Tomatoes	Vegetables	Dried	Turkey	Asia	7/6/2021	2021	Absence
**69**	Sun-Dried Tomatoes	Vegetables	Dried	Turkey	Asia	16/6/2021	2021	Absence
**70**	Sun-Dried Tomatoes	Vegetables	Dried	Turkey	Asia	24/6/2021	2021	Absence
**71**	Sun-Dried Tomatoes	Vegetables	Dried	Turkey	Asia	29/6/2021	2021	Absence
**72**	Strawberries	Soft Fruits/ Berries	Fresh	Greece	Europe	8/7/2021	2021	Absence
**73**	Cherries	Fruits	Fresh	Greece	Europe	8/7/2021	2021	Absence
**74**	Sun-Dried Tomatoes	Vegetables	Dried	Turkey	Asia	30/8/2021	2021	Absence
**75**	Strawberries	Soft Fruits/ Berries	Fresh	Greece	Europe	31/8/2021	2021	Absence
**76**	Cherries	Fruits	Fresh	Greece	Europe	31/8/2021	2021	Absence
**77**	Sun-Dried Tomatoes	Vegetables	Dried	Turkey	Asia	2/9/2021	2021	Absence
**78**	Sun-Dried Tomatoes	Vegetables	Dried	Turkey	Asia	3/9/2021	2021	Absence
**79**	Sun-Dried Tomatoes	Vegetables	Dried	Turkey	Asia	22/9/2021	2021	Absence
**80**	Sun-Dried Tomatoes	Vegetables	Dried	Turkey	Asia	23/9/2021	2021	Absence
**81**	Sun-Dried Tomatoes	Vegetables	Dried	Turkey	Asia	8/10/2021	2021	Absence
**82**	Sun-Dried Tomatoes	Vegetables	Dried	Turkey	Asia	23/11/2021	2021	Absence
**83**	Sun-Dried Tomatoes	Vegetables	Dried	Turkey	Asia	24/11/2021	2021	Absence
**84**	Sun-Dried Tomatoes	Vegetables	Dried	Turkey	Asia	24/11/2021	2021	Absence
**85**	Sun-Dried Tomatoes	Vegetables	Dried	Turkey	Asia	24/11/2021	2021	Absence
**86**	Sun-Dried Tomatoes	Vegetables	Dried	Turkey	Asia	21/1/2022	2022	Absence
**87**	Sun-Dried Tomatoes	Vegetables	Dried	Turkey	Asia	24/1/2022	2022	Absence
**88**	Sun-Dried Tomatoes	Vegetables	Dried	Turkey	Asia	31/1/2022	2022	Absence
**89**	Sun-Dried Tomatoes	Vegetables	Dried	Turkey	Asia	10/2/2022	2022	Absence
**90**	Raspberries	Soft Fruits/ Berries	Frozen	China	Asia	21/2/2022	2022	Absence
**91**	Raspberries	Soft Fruits/ Berries	Frozen	Serbia	Europe	21/2/2022	2022	Absence
**92**	Sun-Dried Tomatoes	Vegetables	Dried	Turkey	Asia	8/3/2022	2022	Absence
**93**	Sun-Dried Tomatoes	Vegetables	Dried	Turkey	Asia	14/3/2022	2022	Absence
**94**	Sun-Dried Tomatoes	Vegetables	Dried	Turkey	Asia	23/3/2022	2022	Absence
**95**	Sun-Dried Tomatoes	Vegetables	Dried	Turkey	Asia	23/3/2022	2022	Absence
**96**	Sun-Dried Tomatoes	Vegetables	Dried	Turkey	Asia	28/3/2022	2022	Absence
**97**	Sun-Dried Tomatoes	Vegetables	Dried	Turkey	Asia	31/3/2022	2022	Absence
**98**	Sun-Dried Tomatoes	Vegetables	Dried	Turkey	Asia	11/4/2022	2022	Absence
**99**	Sun-Dried Tomatoes	Vegetables	Dried	Turkey	Asia	14/4/2022	2022	Absence
**100**	Sun-Dried Tomatoes	Vegetables	Dried	Turkey	Asia	14/4/2022	2022	Absence
**101**	Sun-Dried Tomatoes	Vegetables	Dried	Turkey	Asia	11/4/2022	2022	Absence
**102**	Sun-Dried Tomatoes	Vegetables	Dried	Greece	Europe	27/4/2022	2022	Absence
**103**	Sun-Dried Tomatoes	Vegetables	Dried	Turkey	Asia	16/5/2022	2022	Absence
**104**	Sun-Dried Tomatoes	Vegetables	Dried	Turkey	Asia	7/6/2022	2022	Absence
**105**	Sun-Dried Tomatoes	Vegetables	Dried	Turkey	Asia	8/6/2022	2022	Absence
**106**	Sun-Dried Tomatoes	Vegetables	Dried	Turkey	Asia	10/6/2022	2022	Absence
**107**	Sun-Dried Tomatoes	Vegetables	Dried	Turkey	Asia	15/6/2022	2022	Absence
**108**	Strawberries	Soft Fruits/ Berries	Fresh	Greece	Europe	29/6/2022	2022	Absence
**109**	Cherries	Fruits	Fresh	Greece	Europe	27/6/2022	2022	Absence
**110**	Sun-Dried Tomatoes	Vegetables	Dried	Turkey	Asia	29/6/2022	2022	Absence
**111**	Sun-Dried Tomatoes	Vegetables	Dried	Turkey	Asia	18/7/2022	2022	Absence
**112**	Sun-Dried Tomatoes	Vegetables	Dried	Turkey	Asia	22/8/2022	2022	Absence
**113**	Sun-Dried Tomatoes	Vegetables	Dried	Turkey	Asia	14/9/2022	2022	Absence
**114**	Sun-Dried Tomatoes	Vegetables	Oil-Based	Turkey	Asia	26/9/2022	2022	Absence
**115**	Sun-Dried Tomatoes	Vegetables	Oil-Based	Turkey	Asia	4/10/2022	2022	Absence

**116**	Sun-Dried Tomatoes	Vegetables	Oil-Based	Turkey	Asia	10/10/2022	2022	Absence
**117**	Dates	Fruits	Dried	Israel	Middle East	17/10/2022	2022	Absence
**118**	Sun-Dried Tomatoes	Vegetables	Dried	Turkey	Asia	17/10/2022	2022	Absence
**119**	Sun-Dried Tomatoes	Vegetables	Oil-Based	Turkey	Asia	25/10/2022	2022	Absence
**120**	Sun-Dried Tomatoes	Vegetables	Oil-Based	Turkey	Asia	1/11/2022	2022	Absence
**121**	Sun-Dried Tomatoes	Vegetables	Dried	Turkey	Asia	15/12/2022	2022	Absence
**122**	Sun-Dried Tomatoes	Vegetables	Dried	Turkey	Asia	9/1/2023	2023	Absence
**123**	Sun-Dried Tomatoes	Vegetables	Dried	Turkey	Asia	10/1/2023	2023	Absence
**124**	Sun-Dried Tomatoes	Vegetables	Dried	Turkey	Asia	19/1/2023	2023	Absence
**125**	Sun-Dried Tomatoes	Vegetables	Oil-Based	Turkey	Asia	20/1/2023	2023	Absence
**126**	Sun-Dried Tomatoes	Vegetables	Dried	Turkey	Asia	26/1/2023	2023	Absence
**127**	Sun-Dried Tomatoes	Vegetables	Oil-Based	Turkey	Asia	12/1/2023	2023	Absence
**128**	Sun-Dried Tomatoes	Vegetables	Dried	Turkey	Asia	7/2/2023	2023	Absence
**129**	Sun-Dried Tomatoes	Vegetables	Dried	Turkey	Asia	6/3/2023	2023	Absence
**130**	Strawberries	Soft Fruits/ Berries	Fresh	Greece	Europe	7/3/2023	2023	Absence
**131**	Strawberries	Soft Fruits/ Berries	Fresh	Greece	Europe	13/3/2023	2023	Absence
**132**	Strawberries	Soft Fruits/ Berries	Fresh	Greece	Europe	16/3/2023	2023	Absence
**133**	Sun-Dried Tomatoes	Vegetables	Oil-Based	Turkey	Asia	22/3/2023	2023	Absence
**134**	Sun-Dried Tomatoes	Vegetables	Dried	Turkey	Asia	22/3/2023	2023	Absence
**135**	Sun-Dried Tomatoes	Vegetables	Dried	Greece	Europe	23/3/2023	2023	Absence
**136**	Sun-Dried Tomatoes	Vegetables	Dried	Turkey	Asia	5/4/2023	2023	Absence
**137**	Sun-Dried Tomatoes	Vegetables	Oil-Based	Turkey	Asia	10/4/2023	2023	Absence
**138**	Sun-Dried Tomatoes	Vegetables	Oil-Based	Turkey	Asia	25/4/2023	2023	Absence
**139**	Strawberries	Soft Fruits/ Berries	Fresh	Greece	Europe	26/4/2023	2023	Absence
**140**	Sun-Dried Tomatoes	Vegetables	Dried	Turkey	Asia	22/5/20123	2023	Absence
**141**	Sun-Dried Tomatoes	Vegetables	Dried	Turkey	Asia	6/6/2023	2023	Absence
**142**	Strawberries	Soft Fruits/ Berries	Frozen	Egypt	Africa	7/6/2023	2023	Absence
**143**	Strawberries	Soft Fruits/ Berries	Fresh	Greece	Europe	9/6/2023	2023	Absence
**144**	Sun-Dried Tomatoes	Vegetables	Dried	Turkey	Asia	14/6/2023	2023	Absence
**145**	Sun-Dried Tomatoes	Vegetables	Oil-Based	Turkey	Asia	22/6/2023	2023	Absence
**146**	Strawberries	Soft Fruits/ Berries	Fresh	Greece	Europe	13/7/2023	2023	Absence
**147**	Sauces	Processed Foods	Fresh	Greece	Europe	13/7/2023	2023	Absence
**148**	Sun-Dried Tomatoes	Vegetables	Dried	Turkey	Asia	13/7/2023	2023	Absence
**149**	Sun-Dried Tomatoes	Vegetables	Oil-Based	Turkey	Asia	13/7/2023	2023	Absence
**150**	Berries	Soft Fruits/ Berries	Dried	Turkey	Asia	25/9/2023	2023	Absence
**151**	Sun-Dried Tomatoes	Vegetables	Dried	Turkey	Asia	10/10/2023	2023	Absence
**152**	Sun-Dried Tomatoes	Vegetables	Dried	Turkey	Asia	13/10/2023	2023	Absence
**153**	Sun-Dried Tomatoes	Vegetables	Dried	Turkey	Asia	1/11/2023	2023	Absence
**154**	Strawberries	Soft Fruits/ Berries	Fresh	Greece	Europe	21/11/2023	2023	Absence
**155**	Tomatoes (Tomatoes, Cherry, Physalis Spp.)	Vegetables	Fresh	Greece	Europe	6/12/2023	2023	Absence
**156**	Strawberries	Soft Fruits/ Berries	Fresh	Greece	Europe	10/1/2024	2024	Absence
**157**	Blackberries	Soft Fruits/ Berries	Fresh	Greece	Europe	10/1/2024	2024	Absence
**158**	Raspberries	Soft Fruits/ Berries	Fresh	Greece	Europe	10/1/2024	2024	Absence
**159**	Sun-Dried Tomatoes	Vegetables	Oil-Based	Turkey	Asia	19/1/2024	2024	Absence
**160**	Sun-Dried Tomatoes	Vegetables	Oil-Based	Turkey	Asia	19/1/2024	2024	Absence
**161**	Sun-Dried Tomatoes	Vegetables	Dried	Turkey	Asia	19/1/2024	2024	Absence
**162**	Sun-Dried Tomatoes	Vegetables	Oil-Based	Turkey	Asia	25/1/2024	2024	Absence
**163**	Blueberries	Soft Fruits/ Berries	Fresh	Greece	Europe	16/2/2024	2024	Absence
**164**	Sun-Dried Tomatoes	Vegetables	Oil-Based	Turkey	Asia	16/2/2024	2024	Absence
**165**	Sun-Dried Tomatoes	Vegetables	Dried	Turkey	Asia	26/2/2024	2024	Absence

**166**	Sun-Dried Tomatoes (Paste)	Processed Foods	Fresh	Greece	Europe	26/3/2024	2024	Absence
**167**	Strawberries	Soft Fruits/ Berries	Frozen	Egypt	Africa	1/4/2024	2024	Absence
**168**	Plums	Fruits	Dried	Chile	America	1/4/2024	2024	Absence
**169**	Sun-Dried Tomatoes	Vegetables	Oil-Based	Turkey	Asia	8/4/2024	2024	Absence
**170**	Raspberries	Soft Fruits/ Berries	Fresh	Greece	Europe	9/4/2024	2024	Absence
**171**	Sun-Dried Tomatoes (Paste)	Processed Foods	Fresh	Greece	Europe	26/4/2024	2024	Absence
**172**	Raspberries	Soft Fruits/ Berries	Fresh	Greece	Europe	14/5/2024	2024	Absence
**173**	Sun-Dried Tomatoes	Vegetables	Dried	Turkey	Asia	15/5/2024	2024	Absence
**174**	Strawberries	Soft Fruits/ Berries	Fresh	Greece	Europe	29/5/2024	2024	Absence
**175**	Cranberries	Soft Fruits/ Berries	Dried	Greece	Europe	3/6/2024	2024	Absence
**176**	Cherries	Fruits	Fresh	Greece	Europe	20/6/2024	2024	Absence
**177**	Apricots	Fruits	Fresh	Greece	Europe	21/6/2024	2024	Absence
**178**	Cherries	Fruits	Fresh	Greece	Europe	24/7/2024	2024	Absence
**179**	Sun-Dried Tomatoes	Vegetables	Fresh	Greece	Europe	6/8/2024	2024	Absence
**180**	Peanuts	Nuts And Seeds	Dried	Argentina	America	2/9/2024	2024	Absence
**181**	Peanuts	Nuts And Seeds	Dried	China	Asia	3/9/2024	2024	Absence
**182**	Sun-Dried Tomatoes	Vegetables	Dried	Turkey	Asia	6/9/2024	2024	Absence
**183**	Sun-Dried Tomatoes	Vegetables	Oil-Based	Turkey	Asia	26/9/2024	2024	Absence
**184**	Garlic	Vegetables	Dried	China	Europe	4/10/2024	2024	Absence
**185**	Processed Fruits	Fruits	Fresh	Greece	Europe	7/10/2024	2024	Absence
**186**	Processed Fruits	Fruits	Fresh	Greece	Europe	7/10/2024	2024	Absence
**187**	Tomato Paste	Processed Foods	Fresh	Greece	Europe	9/10/2024	2024	Absence
**188**	Cherries	Fruits	Fresh	Greece	Europe	16/10/2024	2024	Absence
**189**	Sun-Dried Tomatoes	Vegetables	Dried	Turkey	Asia	23/10/2024	2024	Absence
**190**	Onions	Vegetables	Dried	India	Asia	23/10/2024	2024	Absence
**191**	Hazel	Nuts And Seeds	Dried	Turkey	Asia	25/10/2024	2024	Absence
**192**	Fruits	Fruits	Dried	China	Asia	25/10/2024	2024	Absence
**193**	Beetroots	Vegetables	Dried	China	Asia	25/10/2024	2024	Absence
**194**	Mango	Fruits	Dried	Thailand	Asia	25/10/2024	2024	Absence
**195**	Sun-Dried Tomatoes	Vegetables	Dried	Turkey	Asia	30/10/2024	2024	Absence
**196**	Peanuts	Nuts And Seeds	Dried	Egypt	Africa	7/11/2024	2024	Absence
**197**	Cassius	Nuts And Seeds	Dried	Vietnam	Asia	19/11/2024	2024	Absence
**198**	Sun-Dried Tomatoes	Vegetables	Dried	Greece	Europe	20/11/2024	2024	Absence
**199**	Sun-Dried Tomatoes	Vegetables	Dried	Turkey	Asia	21/11/2024	2024	Absence
**200**	Sun-Dried Tomatoes	Vegetables	Dried	Turkey	Asia	21/11/2024	2024	Absence
**201**	Strawberries	Soft Fruits/ Berries	Fresh	Egypt	Africa	27/11/2024	2024	Absence
**202**	Cinnamon	Others	Dried	Indonesia	Asia	16/12/2024	2024	Absence

**Sample No**	**Type Of Food**	**Type Of Food Categories**	**Kind**	**Origin**	**Origin Categories**	**Order Date**	**Year**	**NoV**
**1**	Okra	Vegetables	Frozen	Greece	Europe	18/12/2019	2019	Absence
**2**	Okra	Vegetables	Frozen	Greece	Europe	18/12/2019	2019	Absence
**3**	Raspberries	Soft Fruits/Berries	Frozen	Greece	Europe	6/2/2020	2020	Absence
**4**	Raspberries	Soft Fruits/Berries	Frozen	Greece	Europe	6/2/2020	2020	Absence
**5**	Mussels	Animal-Based Products	Frozen	Greece	Europe	10/3/2020	2020	Absence
**6**	Strawberries	Soft Fruits/Berries	Fresh	Greece	Europe	30/3/2020	2020	Absence
**7**	Raspberries	Soft Fruits/Berries	Frozen	Greece	Europe	27/4/2020	2020	Absence
**8**	Sun-Dried Tomatoes	Vegetables	Dried	Turkey	Asia	18/5/2020	2020	Absence
**9**	Sun-Dried Tomatoes	Vegetables	Dried	Turkey	Asia	18/5/2020	2020	Absence
**10**	Raspberries	Soft Fruits/Berries	Frozen	Greece	Europe	25/5/2020	2020	Absence
**11**	Strawberries	Soft Fruits/Berries	Frozen	Egypt	Africa	7/7/2020	2020	Absence
**12**	Raspberries	Soft Fruits/Berries	Frozen	Greece	Europe	21/9/2020	2020	Absence

**13**	Fragostafylla	Soft Fruits/Berries	Frozen	Greece	Europe	22/9/2020	2020	Absence
**14**	Fragostafylla	Soft Fruits/Berries	Frozen	Greece	Europe	22/9/2020	2020	Absence
**15**	Sun-Dried Tomatoes	Vegetables	Dried	Turkey	Asia	14/12/2020	2020	Absence
**16**	Sun-Dried Tomatoes	Vegetables	Dried	Turkey	Asia	3/2/2021	2021	Absence
**17**	Strawberries	Soft Fruits/Berries	Fresh	Greece	Europe	12/4/2021	2021	Presence
**18**	Sun-Dried Tomatoes	Vegetables	Dried	Turkey	Asia	4/6/2021	2021	Absence
**19**	Sun-Dried Tomatoes	Vegetables	Dried	Turkey	Asia	29/6/2021	2021	Absence
**20**	Strawberries	Soft Fruits/Berries	Fresh	Greece	Europe	8/7/2021	2021	Presence
**21**	Cherries	Fruits	Fresh	Greece	Europe	8/7/2021	2021	Presence
**22**	Strawberries	Soft Fruits/Berries	Fresh	Greece	Europe	31/8/2021	2021	Absence
**23**	Cherries	Fruits	Fresh	Greece	Europe	31/8/2021	2021	Absence
**24**	Raspberries	Soft Fruits/Berries	Frozen	China	Asia	21/2/2022	2022	Absence
**25**	Raspberries	Soft Fruits/Berries	Frozen	Serbia	Europe	21/2/2022	2022	Absence
**26**	Strawberry	Soft Fruits/Berries	Fresh	Greece	Europe	29/5/2022	2022	Absence
**27**	Cherries	Fruits	Fresh	Greece	Europe	27/6/2022	2022	Absence
**28**	Sun-Dried Tomatoes	Vegetables	Dried	Turkey	Asia	9/1/2023	2023	Absence
**29**	Strawberries	Soft Fruits/Berries	Fresh	Greece	Europe	7/3/2023	2023	Absence
**30**	Strawberries	Soft Fruits/Berries	Fresh	Greece	Europe	13/3/2023	2023	Absence
**31**	Strawberries	Soft Fruits/Berries	Fresh	Greece	Europe	16/3/2023	2023	Absence
**32**	Sun-Dried Tomatoes	Vegetables	Dried	Greece	Europe	23/3/2023	2023	Absence
**33**	Strawberries	Soft Fruits/Berries	Fresh	Greece	Europe	26/4/2023	2023	Absence
**34**	Strawberries	Soft Fruits/Berries	Fresh	Greece	Europe	27/4/2023	2023	Absence
**35**	Strawberries	Soft Fruits/Berries	Fresh	Greece	Europe	9/6/2023	2023	Absence
**36**	Sun-Dried Tomatoes	Vegetables	Fresh	Greece	Europe	13/7/2023	2023	Absence
**37**	Sauces	Processed Foods	Fresh	Greece	Europe	13/7/2023	2023	Absence
**38**	Strawberries	Soft Fruits/Berries	Fresh	Greece	Europe	21/11/2023	2023	Absence
**39**	Tomatoes (Tomatoes, Cherry, Physalis Spp.)	Vegetables	Fresh	Greece	Europe	6/12/2023	2023	Absence
**40**	Strawberries	Soft Fruits/Berries	Fresh	Greece	Europe	10/1/2024	2024	Absence
**41**	Blackberries	Soft Fruits/Berries	Fresh	Greece	Europe	10/1/2024	2024	Absence
**42**	Raspberries	Soft Fruits/Berries	Fresh	Greece	Europe	10/1/2024	2024	Absence
**43**	Blueberries	Soft Fruits/Berries	Fresh	Greece	Europe	16/2/2024	2024	Absence
**44**	Sun-Dried Tomatoes (Paste)	Processed Foods	Fresh	Greece	Europe	26/3/2024	2024	Presence

**45**	Raspberries	Soft Fruits/Berries	Fresh	Greece	Europe	9/4/2024	2024	Absence
**46**	Sun-Dried Tomatoes (Paste)	Processed Foods	Fresh	Greece	Europe	26/4/2024	2024	Absence
**47**	Raspberries	Soft Fruits/Berries	Fresh	Greece	Europe	14/5/2024	2024	Absence
**48**	Strawberries	Soft Fruits/Berries	Fresh	Greece	Europe	29/5/2024	2024	Absence
**49**	Cherries	Fruits	Fresh	Greece	Europe	20/6/2024	2024	Absence
**50**	Apricots	Fruits	Fresh	Greece	Europe	21/6/2024	2024	Absence
**51**	Cherries	Fruits	Fresh	Greece	Europe	24/7/2024	2024	Absence
**52**	Sun-Dried Tomatoes	Vegetables	Fresh	Greece	Europe	6/8/2024	2024	Absence
**53**	Blackberries	Soft Fruits/Berries	Frozen	Serbia	Europe	5/9/2024	2024	Absence
**54**	Tomato Paste	Processed Foods	Fresh	Greece	Europe	9/10/2024	2024	Absence
**55**	Yoghurt	Animal-Based Products	Fresh	Greece	Europe	9/10/2024	2024	Absence
**56**	Yoghurt	Animal-Based Products	Fresh	Greece	Europe	10/10/2024	2024	Absence
**57**	Fish	Animal-Based Products	Other	Greece	Europe	11/10/2024	2024	Absence
**58**	Cherries	Fruits	Fresh	Greece	Europe	16/10/2024	2024	Absence
**59**	Salad	Vegetables	Fresh	Greece	Europe	6/11/2024	2024	Absence
**60**	Chicken	Animal-Based Products	Other	Greece	Europe	6/11/2024	2024	Absence
**61**	Sun-Dried Tomatoes	Vegetables	Dried	Greece	Europe	20/11/2024	2024	Absence
**62**	Lettuce	Vegetables	Fresh	Egypt	Africa	28/11/2024	2024	Absence
**63**	Blueberries	Soft Fruits/Berries	Fresh	Canada	America	29/11/2024	2024	Absence
**64**	Spinach	Vegetables	Frozen	Turkey	Asia	5/12/2024	2024	Absence
